# Supplementary material for: Variations in the Circle of Willis in a large population sample using 3D TOF angiography: The Tromsø Study
Source: PLoS One. 2020 Nov 3;15(11):e0241373. doi: 10.1371/journal.pone.0241373 (PMC7608873; doi:10.1371/journal.pone.0241373)
Supplement: S1 File — (DOCX) [file pone.0241373.s001.docx]

**Supporting information for**

Variations in the Circle of Willis in a large population sample using 3D TOF angiography:
The Tromsø Study

Lars B. Hindenes^1,2^*, Asta K. Håberg^3,4^, Liv Hege Johnsen^1^, Ellisiv B. Mathiesen^1,5^, David Robben^6,7^, Torgil R. Vangberg^1,2^

^1^Department of Clinical Medicine, Faculty of Health Sciences, UiT The Arctic University of Norway, Tromsø, Norway

^2^PET Centre, University Hospital of North Norway, Tromsø, Norway

^3^Department of Radiology and Nuclear Medicine, St. Olav University Hospital, Trondheim, Norway

^4^Department of Neuromedicine and Movement Science, Norwegian University of Science and Technology (NTNU), Trondheim, Norway

^5^Department of Neurology, University Hospital of North Norway, Tromsø, Norway

^6^ESAT-PSI, Department of Electrical Engineering, KU Leuven, Leuven, Belgium

^7^Icometrix, Leuven, Belgium

* Corresponding author

E-mail: lars.b.hindenes@uit.no

**Contents of this file**

S1-5 Figs, S1 Table, supporting Methods and supporting Comments about S2 File (separate xlsx file).

**S1 Fig. 3D volume rendering of a time-of-flight magnetic resonance image depicting a subject’s complete Circle of Willis with artery segment labels.**
Image follows neurological convention, where left is left and right is right. ACA-2: Distal anterior cerebral artery. ACA-1: Proximal anterior cerebral artery. ACoA: Anterior communicating artery. MCA: Middle cerebral artery. ICA: Internal carotid artery. PCoA: Posterior communicating artery. PCA-1: Proximal posterior cerebral artery. PCA-2: Distal posterior cerebral artery. BA: Basilar artery.

**S2 Fig.** **Illustration of the unilateral dual posterior cerebral artery variant.**Only a right-side variant is shown, and may as such be mirrored. The illustrated morphology may occur simultaneously as other deviations from a complete Circle of Willis. PCA = Posterior cerebral artery.

**S3 Fig.** **Back-to-back histograms showing the age distribution of the men and women in the current study.**

**S4 Fig.** **Flowchart illustration of the intra rater test of a subsample of 100 randomly selected subjects.**
There were 21 mismatches in total. Each ellipse represents a variant, and their corresponding dotted arrows denote which “original” variant was misclassified as which in the reclassification. The letters, and in some cases with numbers, respectively denote which arteries were wrongfully classified as missing (plus) or present (minus), and how often the mistake was done. If no number is shown, that indicates that the misclassification happened only once. Each variant’s name is put together by the missing segments with the following notation: O = Complete variant (no missing arteries), Ac = Anterior communicating artery, A = Anterior cerebral artery, Pc = Posterior communicating artery, 2Pc = Bilateral posterior communicating artery, P = Posterior cerebral artery, B = Basilar artery, while the suffixes “r” and “l” denote right and left lateralization of arteries.

**S5 Fig.** **Flowchart illustration of the inter rater test.**
The subsample of 100 randomly selected subjects is the same as in the intra rater test. There were 18 mismatches in total. Each ellipse represents a variant, and their corresponding dotted arrows denote which “original” variant was misclassified as which in the reclassification. The letters, and in some cases with numbers, respectively denote which arteries were wrongfully classified as missing (plus) or present (minus), and how often the mistake was done. If no number is shown, that indicates that the misclassification happened only once. Multiple wrongfully classified arteries per mistake are denoted by a combination of letters and the “&”. Each variant’s name is put together by the missing segments with the following notation: O = Complete variant (no missing arteries), Ac = Anterior communicating artery, A = Anterior cerebral artery, Pc = Posterior communicating artery, 2Pc = Bilateral posterior communicating artery, P = Posterior cerebral artery, 2P = Bilateral posterior cerebral artery, M = Middle cerebral artery, B = Basilar artery, while the suffixes “r” and “l” denote right and left lateralization of arteries.

**S1 Table.** Subjects with rare Circle of Willis variants [Number of cases (Percentage of total)].

| Variant: | Number of subjects: |
| --- | --- |
| Ar | 9 (0.5) |
| AlPl | 7 (0.4) |
| AlPcrPl | 5 (0.3) |
| ArPcr | 5 (0.3) |
| Ar2P | 4 (0.2) |
| ArPr | 4 (0.2) |
| Al | 3 (0.2) |
| AlPcr | 3 (0.2) |
| ArPcrPl | 3 (0.2) |
| AlPcl | 2 (0.1) |
| 2AAc2Pc | 1 (0.1) |
| 2MAlPcr | 1 (0.1) |
| AcPlB | 1 (0.1) |
| AcPrB | 1 (0.1) |
| AlPclPr | 1 (0.1) |
| AlPr | 1 (0.1) |
| ArAc2Pc | 1 (0.1) |
| Il | 1 (0.1) |
| Il2Pc | 1 (0.1) |
| IlPcr | 1 (0.1) |
| Ir | 1 (0.1) |
| IrAr | 1 (0.1) |
| Ml2Pc | 1 (0.1) |
| MrAr | 1 (0.1) |
| PclPrB | 1 (0.1) |

Each variant’s name is put together by the missing segments with the following notation: 2A = Bilateral anterior cerebral artery, Ac = Anterior communicating artery, 2Pc = Bilateral posterior communicating artery, 2M = Bilateral middle cerebral artery, A = Anterior cerebral artery, Pc = Posterior communicating artery, P = Posterior cerebral artery, B = Basilar artery, 2P = Bilateral posterior cerebral artery, I = Internal carotid artery, M = Middle cerebral artery, while the suffixes “r” and “l” denote right and left lateralization of arteries.

# Supporting Methods

## Conditional probability/frequency estimates

Conditional probabilities or frequencies were calculated using the following equation:

$$P\left( A \mid B \right)=\frac{P(A\cap B)}{P(B)},$$

where $A$ and $B$ denote missing arterial segments in the CoW. $P\left( A \mid B \right)$ is the probability of missing artery $A$ given that artery$B$ is missing, while $P(A\cap B)$ is the joint probability or frequency estimate, i.e. the probability that both artery $A$ and $B$ are missing at the same time. Lastly, $P(B)$ is the probability of missing artery $B$.

## Joint probability/frequency estimates

The joint probability defined as $P(A\cap B)$, is calculated by:

$$P\left( A\cap B \right)=\frac{Number of times both A and B are missing in a variant}{Total number of variants},$$

where $A$ and $B$ represent different arteries.

## Probability/frequency estimates

The probability of missing artery $B$, $P(B)$, is calculated by:

$$P\left( B \right)=\frac{Number of variants with a missing artery B}{Total number of variants},$$

where $B$ denotes an artery that is missing. Individual variant frequencies are calculated similarly.

## Chi-squared test, Cochran-Mantel-Haenszel test and degrees of freedom

The notation used to denote the dimensions of the Chi-squared test $R\times C$ is used to specify which part of the data tables that is used. Here $R$ denotes the number of rows, while $C$ denotes the number of columns. In Chi-squared tests, $\left( C-1 \right)\cdot(R-1)$ is the degree of freedom used to specify the quantiles. As such, it is possible to determine if a test is significant or not, avoiding ambiguity during retesting. Similarly, in the Cochran-Mantel-Haenszel tests we used the notation $R\times C\times L$ to specify which part of the data is used. $R$ and $C$ still denote row and columns respectively, and the $L$ denote the dimension of the nominal variable that is controlled for. As in Chi-squared tests, the degree of freedom for a Cochran-Mantel-Haenszel test is $\left( C-1 \right)\cdot(R-1)$ when both the row and column is a nominal variable.

## Selecting the minimum threshold separating common and uncommon variants from rare variants in Chi-squared test and Cochran-Mantel-Haenszel tests

To perform valuable statistical inference and to include as many variants as possible, we chose a threshold separating the common and the other rare Circle of Willis variants. This threshold was chosen with consideration to the binomial test assuming the homogeneous null hypothesis case when the probability of both cases is 50%, i.e. equally likely and no significant heterogeneity between the two options. Mathematically this binomial test scenario can be written as:

$$H_{0}:p=0.50$$

$$H_{1}:p\neq0.50$$

If we look up this binomial cumulative distribution with the respective null hypothesis in a table of quantiles or statistical software (“qbinom” command in R), we find that a minimum of 10 observations can return p-values to a minimum of P = 0.001 in binomial tests. Such a minimum p-value still has the possibility to yield significant values with respect to a standard significance level and significance levels corrected for a few tests. Although the Chi-squared test and the Cochran-Mantel-Haenszel test are not binomial tests, we do look at differences between dichotomic groupings of variants on a more general (multinomial) level, including the addition of a control variable, and the concept can therefore translate sufficiently for the purpose of thresholding.

## Validity of Cochran-Mantel-Haenszel test

We did Woolf tests to determine whether the Cochran-Mantel-Haenszel test was appropriate to test our hypotheses of conditional independence between Circle of Willis variant frequencies and sex, and mean-split age, while controlling for the other. In other words, age was controlled for when testing variants and sex, and sex was controlled for when testing variants and age. The Woolf test tests whether the three-way association between the three nominal variables is significant, and if it is insignificant, then the Cochran-Mantel-Haenszel test is appropriate to use. We provided two 3D tables ($23\times2\times2$), interchanging sex and mean-split age between the two tables, and the results were both equal to $X^{2}$(1, N = 1,864) = 0.0014 with P = .9697. This implied no three-way association, and the Cochran-Mantel-Haenszel tests were appropriate to use.

# Supporting Comments

Alongside this supplementary section there is an additional Supplementary Spreadsheet S1 (xlsx) file that contains a comparison with the only other Circle of Willis study with a sample higher than ours, that used time-of-flight angiography and had a classification scheme possible to compare the Circle of Willis variants without substantial loss.

The translation of the other study’s CoW variants, to our Circle of Willis nomenclature, went without substantial issues. Possible issues were that they had classified fetal-type posterior (FTP) variants and a dual posterior cerebral artery variant, which we had not considered as unique enough variants within our classification scheme. Nonetheless, we handled these variants using our three classification criteria defined in the main text. Further, to enable direct comparison between variants in our study and the other study, we had to remove lateralization in our CoW variants and omit a miniscule amount of rare CoW variants that we could not compare. The numerical details of the comparison can be found in the (xlsx) file “S2_file.xlsx” alongside corresponding comments to understand the comparison.
